# A Cure for Leprosy at Last

**Published:** 1924-03

**Authors:** 


### A CURE FOR LEPROSY AT LAST.

A meeting in support of the British Empire Leprosy Relief Association was held on January 31, at the Mansion House, the Lord Mayor presiding. The Association is appealing for £250,000 to extend the cure for leprosy which is believed to have been found, and also to continue research work. The speakers were Lord Chelmsford, Chairman of the Association, the Duke of Devonshire, Sir Humphrey Rolleston, President of the Royal College of Physicians, Sir Leonard Rogers, Hon. Medical Secretary of the Association, Mr. Frank Oldrieve, the Secretary, and Sir Sydney Olivier, Secretary of State for India. The Lord Mayor read a message which had been sent by the Prince of Wales, who is Patron of the Association, in which he said that the elimination of leprosy from the British Empire is a wonderful ideal, which he is confident can be realised if it is accorded the support it merits.

### An Imperial Movement.

Lord Chelmsford, after mentioning that he had received two cheques for £100 towards the amount aimed at from the King and the Prince of Wales, explained that the movement was Imperial in character, for three Government departments—the Foreign Office, the Colonial Office and the India Office—were backing the Association. He pointed out that if leprosy were still a curse in this country money would roll in when it was announced that a cure had been discovered, but that England could not fail to be moved by the fact that 300,000 lepers existed in the British Empire, and that only 15,000 could be cared for.

### An Example of Preventive Medicine.

Sir Humphrey Rolleston said that our duty as an Empire lay in trying to eliminate this dreadful scourge and set an example of preventive medicine. This country happily was now practically free from the disease, there being only the one small colony of St. Giles, near Chelmsford, which was in urgent need of funds. Sir Humphrey said that doubtless isolation had done much to diminish the spread of the disease, but segregation must be made attractive instead of repulsive, though that alone is not enough to stamp out the disease. He believed that Sir Leonard Rogers had found in the fatty acids of certain oils, and in the preparation called morrhuate of sodium, a remedy that bids fair to be a real cure. Sir Leonard Rogers, after describing his researches into the old Indian cure for leprosy—chaulmoogra oil—said that in it he had found a remedy which by intravenous injections destroyed the leprous bacillus. Mentioning numbers, he stated that of the cases in which injection had been continued for the necessary periods of 6 to 9 months, 74 per cent, and after 12 to 15 months 93 per cent. showed definite improvement, and quite a number had been definitely cleared up. There was good reason to believe therefore that permanent cures could be effected. There was still, of course, an unlimited field for research, and there were possibilities of discovering a more rapid cure and possibly also one for tuberculosis. Mr. F. Oldrieve, speaking of the tremendous hope that had dawned for the lepers, said that leprosy could be stamped out in 30 or 40 years, providing the necessary money could be raised.

### TWO HOSPITALS MERGE THEIR MEDICAL STAFFS.

An interesting arrangement has been made between the Queen's Hospital, Birmingham, and the Birmingham and Midland Hospital for Diseases of the Nervous System. Without amalgamating, the medical and nursing staffs are being united, so that, in other words, in respect to medical treatment and nursing, the Queen's Hospital will be responsible for both institutions. The object is to give to the smaller hospital the benefit of the greater resources of the larger. To complete the arrangement the Queen's Hospital has appointed a physician for out-patients to work solely at the Midland Hospital. There the out- and in-patient departments are in different places, and admission is by payment. The administration and finance of the two hospitals will remain as before, separate and distinct. Since its foundation in 1914 the special hospital has had many difficulties to meet, and the new association, as the arrangement is called, is probably a wise solution, which time will test in an interesting way. We ourselves have advocated co-ordination for this hospital in the past, and Mr. Joseph Ansell, the chairman, will be relieved at the completion of the negotiations, which, being unusual and perhaps complicated, have taken many months to complete.

